# Barriers and Strategies for Recruiting Care Partners During the Hospitalization of People Living with Dementia: Lessons from a Hospital-Based Randomized Controlled Trial

**DOI:** 10.3390/ijerph23040447

**Published:** 2026-03-31

**Authors:** Te-Lien Ku, Kayla Dillon, Henry Karelitz, Anne Mortensen, Shelly C. VanDenBergh, Dani M. Edwards, Emmanuel Quarcoo, Courtney M. Kuhlman, Molly M. Gerhardt, Élise N. Arsenault Knudsen, Beth Fields

**Affiliations:** 1 Clinical Practice, Innovation, and Research Division, School of Pharmacy, University of Wisconsin-Madison, Madison, WI 53705, USA; tku6@wisc.edu; 2 Department of Kinesiology, University of Wisconsin-Madison, Madison, WI 53706, USA; kedillon@wisc.edu (K.D.); karelitz@wisc.edu (H.K.); amortensen3@wisc.edu (A.M.); 3 UW Health University Hospital, Madison, WI 53792, USA; svandenbergh@uwhealth.org (S.C.V.); dedwards@uwhealth.org (D.M.E.); equarcoo@uwhealth.org (E.Q.); ckuhlman@uwhealth.org (C.M.K.); 4 UW Health East Madison Hospital, Madison, WI 53718, USA; mgerhardt@uwhealth.org; 5 School of Nursing, University of Wisconsin-Madison, Madison, WI 53706, USA; arsenaultknu@wisc.edu

**Keywords:** care partners, Alzheimer’s disease and related dementias, hospital, recruitment, social marketing framework, caregiver-centered care

## Abstract

**Highlights:**

**Public health relevance—How does this work relate to a public health issue?**

- In the United States, care partners of hospitalized people living with dementia experience substantial challenges, including poor preparedness, high burden, and depressive symptom.

**Public health significance—Why is this work of significance to public health?**

- There is an urgent public health need to systematically identify and support care partners of people living with dementia during hospitalization, as they play a central role in coordinating post-discharge care but are often overlooked in acute care settings.
- Improving care partner recruitment in hospital-based research and interventions is critical to public health, enabling timely engagement that strengthens care transitions, reduces preventable post-discharge complications, and supports care partner well-being.

**Public health implications—What are the key implications or messages for practitioners, policy makers and/or researchers in public health?**

- Improved recruitment strategies can enhance engagement of care partners of hospitalized patients living with dementia.
- Effective recruitment is essential for implementing evidence-informed interventions that improve care partner outcomes.

**Abstract:**

*Background*: Recruiting care partners (CPs) of hospitalized people living with dementia is challenging due to rapid discharge timelines and complex inpatient workflows. This study identified barriers to CP recruitment encountered during the first year of a hospital-based randomized controlled trial and the strategies implemented in response to them. *Methods*: A qualitative descriptive study using thematic analysis of twelve study coordination and implementation meeting transcripts was conducted. Recruitment outcomes were examined to assess changes before and after implementation of the enhanced recruitment workflow. *Results*: Eight recruitment barriers were identified, including fragmented electronic health record (EHR) documentation, uncertainty in CP presence, limited clinician availability, passive study promotion, and inefficient clinical–research team collaboration. Nine strategies were developed and implemented in response to these barriers. The enhanced recruitment workflow was temporally associated with an increase in average monthly enrollment from 0.25 participants (April–July 2024) to 4 participants (August 2024–December 2025). Over the same periods, the cumulative enrollment rate was 3% and 23%, respectively. *Conclusions*: Care partner recruitment in inpatient settings is highly context-dependent and requires iterative, implementation-informed adaptation. Effective recruitment may be supported by embedding processes into routine inpatient workflows, clarifying recruitment roles, leveraging EHR-supported identification, and maintaining close clinical–research team collaboration to respond to the unpredictable presence of care partners and short discharge windows.

## 1. Introduction

Dementia care partners (CPs), commonly described in the literature as caregivers or carers, are family members or significant others who provide support with daily living and healthcare and are often involved in care coordination and decision-making for individuals living with dementia [1]. Despite the multifaceted nature of their roles, recruiting CPs into clinical and community research remains a complex and resource-intensive process. Prior studies have identified numerous barriers, including limited time and competing responsibilities among potential participants, challenges in engaging underrepresented groups, mistrust due to insufficient or unclear communication about research, stigma related to conditions such as dementia, logistical barriers within healthcare settings, and inconsistencies in recruitment procedures [2,3,4,5,6].

Although strategies such as CP engagement, clinician referrals, patient registries, and multi-platform outreach (e.g., phone calls, mailings, flyers, emails, and social media) have been shown to improve recruitment [3,7,8], most evidence comes from community-based research, with limited attention to clinical environments such as inpatient settings. In these settings, recruitment is further complicated by fast-paced, time-sensitive workflows, high patient turnover, intensive clinical demands, and multidisciplinary coordination among healthcare teams [9]. For individuals living with Alzheimer’s disease and related dementias (ADRD), hospitalization often represents a critical transition in care, during which CPs face urgent decisions regarding treatment, discharge, and long-term care planning. These contextual features can directly influence how CPs are identified, approached, and engaged in clinical and research activities.

With approximately 57 million people worldwide living with ADRD [10]—a number projected to increase substantially—many individuals rely on CPs to maintain quality of life and continuity of care [11,12,13]. This underscores the need for policies that prioritize advances in CP engagement and research. In the United States, the *2022 National Strategy to Support Family Caregivers* highlights this priority through five main goals, including Goal 2: “Advance partnerships and engagement with family caregivers” and Goal 5: “Expand data, research, and evidence-based practices to support family caregivers” [14].

In alignment with this national priority, our team adapted the Care Partner Hospital Assessment Tool for the dementia population to develop the Dementia Care Partner Hospital Assessment Tool (D-CHAT) [15,16]. The D-CHAT is a standardized decision-support tool designed to integrate CPs into hospital care processes by assessing CP support needs at or near patient admission and facilitating tailored consultation and training from hospital clinicians during hospitalization. The paper-based tool is currently being tested in a hospital-based randomized controlled trial to evaluate its feasibility and impact on improving CP preparedness for caregiving, burden, and depression.

During early implementation of the trial, substantial challenges emerged in identifying and enrolling eligible CPs within inpatient workflows. Although CP recruitment in inpatient settings is widely recognized as complex, there remains limited implementation-informed guidance on how to operationalize recruitment workflows for CPs of hospitalized people living with dementia. Effective and context-sensitive recruitment processes are key components of the successful implementation of CP interventions during hospitalization. To address this gap, we conducted a focused process evaluation to identify recruitment barriers, implement an enhanced recruitment workflow, document context-responsive strategies, and provide operational recommendations for future hospital-based caregiving research.

## 2. Materials and Methods

### 2.1. Design

This study employed a qualitative descriptive design using thematic analysis of transcripts from study coordination and implementation meetings involving academic researchers and clinical partners to identify barriers and strategies related to CP recruitment during the early implementation phase of the parent hospital-based randomized controlled trial.

### 2.2. Study Setting and Context

This study was embedded within a hospital-based randomized controlled trial evaluating the D-CHAT. The trial is being conducted across three inpatient units (one family medicine unit and two general internal medicine units) within two hospitals in a single academic medical system in the Midwestern United States. Within the trial, only CPs are enrolled and provide oral informed consent, whereas hospitalized patients living with dementia are not enrolled as research participants. CP eligibility, including confirmation of the care recipient’s dementia diagnosis, is determined through standard screening procedures in accordance with the institutional review board–approved protocol (NCT05592366). Additional details about the trial design and procedures are available in a published protocol [17].

**Figure 1:**
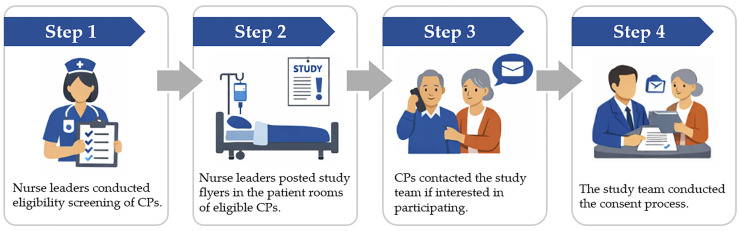
Initial recruitment workflow for the D-CHAT parent study. CP: Care partner.

Both hospitals are large, tertiary-care academic medical centers characterized by high patient turnover, complex care needs, and time-sensitive workflows. Within this context, nurse leaders and hospital coordinators involved in the study balanced recruitment activities with routine clinical responsibilities, including care coordination, staff supervision, and unit-level operations. These competing priorities shaped how recruitment activities were integrated into daily workflows and contributed to the barriers identified in this study. At study initiation, CP recruitment relied on a passive approach. Nurse leaders serving as unit-based D-CHAT implementation champions conducted eligibility screening, posted study flyers in eligible patient rooms, and relied on CPs to initiate contact with the study team. Direct outreach from the study team was minimal during this phase. Figure 1 presents an overview of the initial recruitment workflow.

### 2.3. Study Team Composition

This study involved a collaborative clinical-academic partnership comprising team members with complementary expertise. The team included pre-health undergraduate and graduate students in occupational therapy and pharmacy, researchers with expertise in qualitative methods, implementation science, dementia, and caregiving, and full-time nurse leaders from an academic medical system, each with more than five years of hospital experience and at least a bachelor’s degree. The team was predominantly female and White, and its composition enabled integration of academic rigor, clinical expertise, and practice-based perspectives to inform the ongoing trial.

### 2.4. Data Collection

A total of twelve meetings were conducted between April 2024 and December 2024, including eight virtual full-team meetings involving study team members, nurse leaders, and hospital coordinators responsible for site-level logistics, and four in-person study-team-only meetings. This period was selected to capture both the pre-implementation phase (April–July 2024), when recruitment followed the initial workflow, and the early post-implementation phase (August–December 2024), following introduction of the enhanced recruitment workflow. Meetings supported ongoing study coordination and identification of recruitment- and intervention-related barriers and strategies over time. Each meeting followed a semi-structured agenda that included: (1) study updates on enrollment progress; (2) follow-up on prior actions; and (3) facilitated reflection, during which nurse leaders and study team members responsible for recruitment and intervention activities described barriers encountered and discussed solutions. Meetings were facilitated by the principal investigator or study team manager to ensure consistent coverage and equitable participation.

All meetings were audio-recorded with participant consent and transcribed verbatim by the first author. Transcripts served as the primary qualitative data source, capturing real-time reflections on recruitment challenges and workflow adaptations across pre- and post-implementation phases. Barriers and strategies emerged through naturalistic discussions among participants rather than responses to pre-specified interview questions. The meetings also brought together stakeholders from multiple roles and organizational contexts, allowing collaborative evaluation and refinement of recruitment processes.

### 2.5. Data Analysis

Meeting transcripts constituted the qualitative dataset and were analyzed using a qualitative descriptive approach, as described by Sandelowski [18] and Bradshaw et al. [19], with thematic analysis used to identify patterns related to recruitment barriers and strategies. Data were managed using NVivo 15.

Analysis proceeded in two phases. First, an inductive thematic analysis was conducted to identify recruitment-related barriers and strategies without applying a priori theoretical categories [20,21]. Two analysts (the first and second authors) independently reviewed transcripts, identified meaning units (i.e., segments of one or more sentences), and generated initial codes. Codes were iteratively compared, refined, and grouped into broader themes based on conceptual similarities. In the second phase, the Social Marketing Framework (SMF) was applied deductively as an organizing framework to interpret the inductively generated themes [22,23,24]. The SMF was selected because it provides a structured approach for understanding factors that are related to CP recruitment and engagement in health research while allowing the analysis to remain grounded in the inductively derived findings. Each theme was reviewed in relation to SMF domain definitions and assigned to the domain that best reflected the underlying barrier or strategy. Discrepancies were resolved through team discussion to reach consensus. When needed, a third study team member (corresponding author) facilitated resolution. Analytic decisions were documented to enhance transparency and trustworthiness.

As part of the deductive analysis, in some instances, themes appeared to span multiple SMF domains. For example, challenges related to staff time and availability could reflect increased workload for nurse leaders (aligned with the “managing the price” domain) or inefficiencies in coordination between clinical and research teams (aligned with the “working with partners” domain). In these cases, the research team revisited the original transcript excerpts and considered the context in which the issue was described to determine the primary underlying mechanism. This process also prompted refinement of theme definitions to better distinguish overlapping concepts and ensure alignment with the SMF domains. Through this iterative process, the SMF functioned not only as a categorization framework but also as a tool for interpreting how recruitment barriers and strategies operated within the inpatient context.

### 2.6. Recruitment Outcomes

Recruitment outcomes were summarized using quantitative enrollment metrics from the parent trial, while qualitative insights from meeting transcripts informed interpretation of recruitment barriers and workflow adaptations. Quantitative recruitment outcomes were compared before and after implementation of the enhanced recruitment workflow. The pre-implementation period spanned 1 April to 31 July 2024 (4 months), and the post-implementation period spanned 1 August 2024 to 31 December 2025 (17 months). Both observation periods corresponded to complete calendar months.

Recruitment performance was evaluated using two measures: (1) average monthly enrollment, calculated as the total number of participants enrolled divided by the number of months in the observation period; and (2) cumulative enrollment rate, defined as the cumulative number of enrolled participants divided by the cumulative number of eligible participants. Recruitment outcomes were monitored through December 2025 to capture longer-term recruitment performance following implementation of the enhanced recruitment workflow. The cumulative enrollment rate was updated monthly to reflect enrollment progress over time. Recruitment data were finalized at the end of each month of the ongoing trial. This pre–post comparison allowed for description of recruitment performance before and after process modifications.

### 2.7. Rigor

Several strategies were used to ensure the rigor of the study. Credibility was supported through investigator triangulation with multiple researchers involved in the analysis process, and through inclusion of diverse participant roles (e.g., nurse leaders and study team members) represented in the meeting transcripts [25]. Credibility was further supported by member checking with nurse leaders during a subsequent full-team meeting, where they reviewed the synthesized findings to confirm their alignment with their experiences [26]. Transferability was enhanced by providing detailed descriptions of the study setting and implementation context, allowing readers to assess the applicability of findings to other inpatient settings [27]. Dependability and confirmability were reinforced through transparent documentation of the analytic process, including an audit trail of analytic decisions and team discussions, a clear description of research procedures, and the use of direct quotes from participants to support key findings [28,29].

## 3. Results

The 12 meetings ranged from 18 to 56 min (mean = 36 min), with 2 to 9 attendees. Table 1 summarizes meeting characteristics and attendee roles. Eight recruitment barriers and nine corresponding strategies were identified and organized across five SMF domains. Table 2 presents the identified barriers, corresponding strategies, and illustrative examples. The following sections describe these barriers and strategies and their implications for the recruitment process, supported by participant excerpts.

### 3.1. SMF Domain: Defining and Identifying the Target Audience

#### 3.1.1. Barrier: Fragmented and Non-Standardized EHR Documentation of Dementia and CP Information

At the initial step of eligibility screening, missed or ambiguous documentation of dementia diagnoses in the EHR affected the accuracy of identifying CPs. Dementia was often omitted or vaguely recorded (e.g., “memory loss,” “cognitive decline”) in the problem list, creating uncertainty about patient eligibility for both study team members and nurse leaders.

*“I’ve had quite a few [admitted patients] over the last couple of weeks that have had questionable diagnoses. And I see that you have down on the comments and notes that MCI (mild cognitive decline) and neurodegenerative disease. We had an intellectual disability too, which there was a question about.”*

*(Full-team meeting #7—Nurse leader)*

In some cases, dementia diagnoses were dispersed across different patient charts in the EHR as patients transitioned between healthcare systems, making it difficult to verify diagnostic completeness.

*“So for context, there are people…there’s lots of health systems, for example, if someone has a chart from another health system and they come into [name of hospital] they’re marked as unidentified and then once they’re discharged those two charts get merged, but we don’t know like if they had dementia from the previous chart until the discharge.”*

*(Study team meeting #1)*

#### 3.1.2. Barrier: Limited Research Team Access to Clinical Information Systems

Because the research team members were not formally employed by the health system (i.e., study site), their access to the EHR system was limited to basic patient screening functions, and they were unable to review merged patient charts, clinician notes, or encounter histories where dementia diagnoses might have been documented. This restricted access constrained the team’s ability to locate relevant diagnostic information and accurately assess participant eligibility.

*“So, let me talk to the EHR folks and just ask. I mean, I do think we can search dementia, but I don’t know we still see everything. You know what I mean? I don’t think we see.”*

*(Full-team meeting #6—Study team member)*

#### 3.1.3. Strategy: Standardized EHR-Based Eligibility Screening Protocol

Both study team members and nurse leaders emphasized the importance of consistently using ICD-10 diagnostic codes, the EHR problem list, and emergency contact information as the standard source of reference for identifying patients with dementia and their CPs. Importantly, emergency contact information was used only as an initial screening reference and was not assumed to represent the CP without further verification by nurse leaders or clinical staff. Establishing these processes was viewed as important for reducing variability related to individual staff knowledge, screening preferences, and unit workflows, and for ensuring that eligible patients and their CPs are reliably captured during recruitment.

*“Neurocognitive disorder is still in our list of eligible. If neurocognitive disorder or like any of the ICD-10 dementia related codes are in their problem list, we do determine them to be eligible.”*

*(Full-team meeting #6—Study team member)*

#### 3.1.4. Strategy: Enhanced Accuracy of Dementia Documentation

Ensuring accurate and up-to-date dementia documentation in the EHR served as a preparatory step for recruitment. This involved nurse leaders confirming unclear diagnoses or ambiguous notes with clinicians and regularly updating records to reflect patients’ current clinical status.

*“…how I was doing the screening was that chart search for the word dementia…if it was being picked up everywhere…, I was reaching out to the clinician and asking them to add it if it was appropriate.”*

*(Full-team meeting #6—Nurse leader)*

#### 3.1.5. Strategy: Research Team EHR Navigation Capacity-Building

Building the research team’s capacity to navigate and use the hospital EHR system involved securing technical support and training from the IT department, obtaining access to necessary EHR functions, and learning from the nurse leaders’ approaches to searching and verifying information. Strengthening these skills enabled the team to more efficiently locate dementia diagnoses, emergency contacts, and other key data needed to identify the target population. This strategy focused on optimizing use of available EHR functions within existing access constraints, rather than expanding formal access privileges.

*“…we will follow up with the exact date once we’ve launched…once we get into the EHR, we might just ask one of the units and sit down with one of you to make sure that we’re looking in the right places because none of us have experience with [specific EHR name].”*

*(Full-team meeting #3—Study team member)*

### 3.2. SMF Domain: Managing the Price

#### 3.2.1. Barrier: Competing Clinical Demands and Limited Recruitment Capacity of Nurse Leaders

Limited time and personnel resources in clinical settings were identified as significant barriers to implementing recruitment activities. Nurse leaders frequently described a “lack of time” and feeling “overwhelmed,” perceiving recruitment as additional work outside their clinical duties. They explained that the added tasks of screening patients and enrolling CPs were difficult to manage alongside existing workloads.

*“I just feel like it’s the volume here is beyond my scope. I can’t, like I’ve spent hours on the D-CHAT, lots of hours on the D-CHAT this week, lots of hours back and forth communicating via phone, via email, via trying to coordinate patient or CP availability with the study team, the back and forth there, getting sucked in. Then when I’m in the room talking to the family, it’s not sustainable.”*

*(Full-team meeting #6—Nurse leader)*

Insufficient time and staffing during night shifts, weekends, and holidays limited opportunities to approach eligible CPs in a timely manner, resulting in missed recruitment windows and reduced enrollment efficiency.

*“And if they can’t schedule until later that evening, we’ll communicate back with you to see if that’s doable before moving forward. Because I don’t want to say yes, we’ll enroll you. And then you all be gone or off on the weekend and then not be able to do anything in case they were randomized to D-CHAT.”*

*(Full-team meeting #7—Study team member)*

#### 3.2.2. Strategy: Designated Recruitment Role Accountability

Having a dedicated study team member responsible for daily screening and enrollment ensured consistent coverage of recruitment activities. Each study unit also designated a primary nurse leader to lead recruitment and communication efforts, creating a shared structure that supported continuity and coordination across units.

*“…before we had to rely on [name of nurse leaders] to do the patient chart review, to do the direct approach, and then hand off to our team to do consent. So now we’re able to do a little bit more during that screening process.”*

*(Full-team meeting #4—Study team member)*

### 3.3. SMF Domain: Improving Accessibility

#### 3.3.1. Barrier: Unpredictable CP Availability During Patients’ Hospitalization

Because the D-CHAT is currently delivered in a paper-based format, CP presence in the hospital was an important factor related to whether the assessment could be completed. Barriers included CPs who lived far from the hospital or were managing their own health issues, making hospital visits difficult. Anticipation of imminent patient discharge also affected decisions about when to visit or how long to stay. Finally, the unpredictable and inconsistent timing of CPs’ hospital visits made it challenging for the study team and nurse leaders to recruit participants and administer the D-CHAT, leading to missed opportunities for full participation.

*“Yeah, we’ve just noticed like the last like, I don’t know, four or five people who have enrolled, that’s like when they want us to be present and which is like we’re able to make that work, but it’s just made us start questioning like are we missing some of the eligible people during that typical visiting hour time when people are coming from work.”*

*(Full-team meeting #6—Study team member)*

#### 3.3.2. Strategy: Proactive Monitoring of CP Presence

Frequent checks for CP presence on the hospital unit involved regularly confirming whether a CP was at the bedside or elsewhere on the unit during the patient’s stay. Nurse leaders conducted in-person checks during their shifts and used the EHR messaging function to hand over reminders to on-duty staff to continue these checks after regular hours. This strategy did not assume consistent CP presence, but rather responded to its unpredictability by establishing repeated, proactive checks intended to minimize missed recruitment opportunities.

*“I’m also routinely rounding, like after I leave the flyer, I’ll check in a couple of times throughout the day, just doing my rounds on the unit and poke my head in to see if there’s any family there.”*

*(Full-team meeting #2—Nurse leader)*

### 3.4. SMF Domain: Promoting the Study

#### 3.4.1. Barrier: Low-Intensity Recruitment Outreach Mechanisms

Limited exposure to the study among CPs posed a barrier to recruitment. Opportunities to learn about the study were constrained by the narrow range of outreach strategies and communication channels used. Study flyers served as the primary recruitment material, but their passive display within hospital units provided minimal opportunity to engage CPs or explain the study’s purpose. As a result, the study’s visibility remained low, and opportunities to clarify eligibility or address questions were limited.

*“…I left the flyer with them and then just said I would be back later in the day. And I connected with them later in the day and they hadn’t had a chance to really review the flyer yet. And then circled back one more time at the end of the day and they were gone. But then connected with them in the morning, and they had decided not to participate.”*

*(Full-team meeting #7—Nurse leader)*

#### 3.4.2. Barrier: Institutional Policy Constraints on Direct Research Engagement

Institutional policies governing research communication and outreach constrained efforts to promote the study to CPs. Direct contact between the study team and CPs was prohibited, requiring nurse leaders to make the initial approach, which delayed engagement.

*“…because we’re not affiliated with [name of hospital]. We tried. If I have a clinical role, and I was in the [name of academic institution] with like an appointment with [name of hospital], like a lot of other investigators have, then we could.”*

*(Full-team meeting #3—Study team member)*

Hospital policy on where study materials could be displayed—allowing flyers only in patient rooms or study units—also limited broader study visibility in public areas and reduced opportunities for CPs to learn about or express interest in participation.

*“I actually saw just yesterday a flyer outside of [unit] and I took it down because I’m like, they’re not going to be able to carry this out just anywhere.”*

*(Full-team meeting #1—Nurse leader)*

#### 3.4.3. Strategy: Enhanced CP Engagement and Study Visibility

Strategies to increase CP awareness and interest in the study included placing study flyers in patient rooms to improve visibility and having nurse leaders proactively reach out to CPs—either in person or by telephone—using a structured script to introduce the study. Care team members also reminded CPs to review the flyer when they were present on the unit.

*“…if you have time and there is someone eligible and they’re not physically present and you can call, that would be wonderful…and then just leave our study phone number, which is on the script that you all have.”*

*(Full-team meeting #3—Study team member)*

### 3.5. SMF Domain: Working with Partners

#### 3.5.1. Barrier: Limited Frontline Staff Awareness and Engagement

Lack of awareness among unit staff not directly involved in the study posed a barrier to recruitment. Many staff members were unfamiliar with the study’s purpose or the D-CHAT, making it difficult for them to introduce or explain the study to CPs or even remind families about the flyers posted in patient rooms. Nursing staff turnover further contributed to this issue, as newly hired employees were often unaware of the study and did not receive consistent orientation or updates regarding the study, reducing opportunities for engagement and referral.

*“And you know as people come and go because it’s going to be what three years, so we will need to keep recycling this as we go forward because we’ll keep getting new people in and um people leaving through normal you know reasons or whatnot, so I think that’ll be important.”*

*(Full-team meeting #1—Nurse leader)*

#### 3.5.2. Barrier: Inconsistent Communication Channels Between Research and Clinical Teams

Inefficient and fragmented communication between the study team and nurse leaders hindered timely and accurate information exchange during recruitment. The inconsistent use of the hospital EHR as a communication tool contributed to this challenge—nurse leaders varied in their use of the EHR as the primary channel for sharing recruitment information, while the study team lacked access to the system outside the hospital and was unfamiliar with certain EHR communication functions. These inconsistencies resulted in unclear reporting and the need for repeated clarification between the two sides, reducing efficiency and hindering the recruitment process.

*“[Nurse leader A] loves Secure Chat [Instant messaging within the EHR], [Nurse leader B] doesn’t look at it enough. She full out said that in one of the email back and forth that we had a couple months ago probably, yeah, um, [Nurse leader C] was not a fan of the Secure Chat but she’s gone now and [Nurse leader D] was just vague, so, um, it’s mixed just whether they even like it.”*

*(Study team meeting #2)*

#### 3.5.3. Strategy: Strengthened Clinician Engagement and Buy-In

To enhance buy-in from nursing staff, we placed posters with an image of the study flyer and additional information in staff break rooms, offered caregiving-focused educational sessions, and promoted the study during unit professional development meetings. Small tokens of appreciation were provided to nursing staff to support team engagement, and additional funding was sought to meet unit-level discretionary needs.

*“…we’ve come and just presented briefly at the professional development days that have been scheduled for this month. I think there’s two left that we’ll also come to just, again, to raise awareness about what we’re doing and make sure everyone’s on the same page.”*

*(Full-team meeting #4—Study team member)*

#### 3.5.4. Strategy: Structured Research-Clinical Communication Pathways

Facilitating communication between the research team and nurse leaders involved holding monthly check-in meetings to share study updates, address concerns, and gather feedback, while cultivating a positive communication climate to support ongoing engagement. We established standardized communication channels to streamline information exchange, including weekly emails summarizing recruitment progress and structured real-time emails for immediate recruitment and enrollment needs. A shared screening log was also created to improve transparency and coordination in recruitment tracking. All communication and documentation were conducted through a university-approved secure email account to ensure data safety.

*“…what has happened that week. And we just have it like in our email record. So, if down the road, you know, we need to refer back to something we have in writing too, to let us know if anything comes up that you have questions about that may have happened like in previous weeks. And we can look through that.”*

*(Full-team meeting #7—Study team member)*

#### 3.5.5. Strategy: Enhanced Cross-Unit Nurse Leader Coordination

Strategies to strengthen collaboration between nurse leaders included using a shared tracking sheet to monitor daily patient admissions and CP status and covering for each other during absences. Nurse leaders across units also shared experiences during monthly meetings and learned from one another’s recruitment strategies, further supporting consistency in recruitment efforts.

*“Yeah, at least for me, [Name] helped create like a list for both of us to use or EHR list to say who the patient is, when they were admitted…So, I can go back really easily to see who was admitted within the last two days and get that up to date.”*

*(Full-team meeting #1—Nurse leader)*

### 3.6. Enhanced Recruitment Workflow and Outcomes

**Figure 2:**
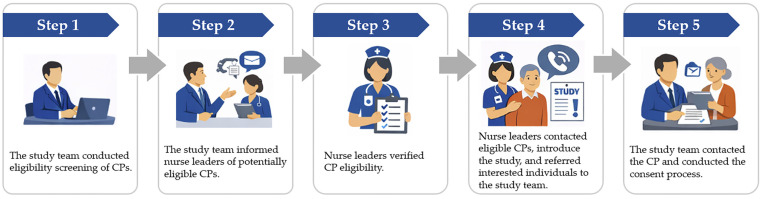
Enhanced recruitment workflow for the D-CHAT parent study (after implementing recruitment strategies). CP: Care partner.

During the implementation of recruitment strategies, the recruitment workflow was refined to support more proactive and coordinated clinician-led engagement. As illustrated in Figure 2, the enhanced workflow included study team–led eligibility screening, nurse leader verification of CP eligibility, introduction of the study to CPs by nurse leaders, and a streamlined referral process for consent.

**Figure 3:**
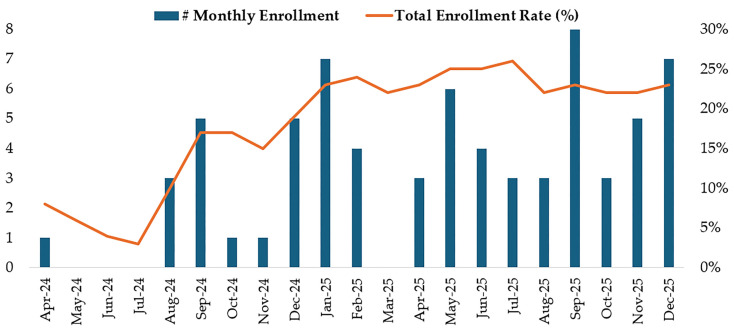
Monthly enrollment and cumulative enrollment rate before and after recruitment strategy implementation (Implementation Initiated in August 2024).

Changes in average monthly enrollment and cumulative enrollment rate pre- and post-implementation are shown in Figure 3. Average monthly enrollment was 0.25 participants per month in the pre-implementation period (April–July 2024) and 4 participants per month in the post-implementation period (August–December 2025). Over the same periods, the cumulative enrollment rate was 3% and 23%, respectively.

## 4. Discussion

This study presents barriers to recruiting dementia CPs in inpatient settings and strategies implemented to support recruitment processes during the first year of a clinical trial. By organizing the inductively identified themes within the SMF domains, we provide conceptual structure to the eight barriers and nine strategies identified. While the study was not designed to compare the effectiveness of individual recruitment strategies, our qualitative analysis of implementation meeting data suggested that improvements in recruitment occurred alongside a set of interrelated workflow modifications—particularly those that strengthened EHR-based case identification, clarified role accountability between the research and clinical teams, and streamlined communication processes within the constraints of clinical priorities and hospital policy. Rather than functioning as isolated high-yield tactics, these adjustments appeared to support greater overall system coherence in a fast-paced inpatient environment. These findings highlight the unique context of hospitals—including fast-paced workflows, short patient stays, unpredictable CP presence, and the need for multidisciplinary teamwork—and offer insight into how recruitment workflows may be adapted to support the feasibility and success of recruitment in hospital-based clinical research. Because multiple recruitment strategies and workflow modifications were introduced concurrently, this study cannot determine the relative contribution of individual strategies to recruitment outcomes.

Our findings highlighted fragmented and inconsistent dementia documentation within the EHR system as a key recruitment barrier. A key feature related to CP recruitment in inpatient settings is the reliance on the EHR to quickly and accurately identify eligible patients and their CPs. Since the study enrollment process can only start after a care recipient is hospitalized—an event that is often unplanned—the inpatient setting lacks opportunities for proactive outreach strategies commonly used in community-based research, such as engaging CP support groups or partnering with community organizations to pre-identify CPs [3,30,31,32]. This unpredictability makes the EHR a vital tool for case identification in inpatient settings, where timely recognition of eligible CPs is crucial [33]. Consistent with prior research highlighting gaps in patient and CP documentation as a recruitment barrier, we found CP information scattered across non-standardized fields, with dementia diagnoses documented inconsistently or incompletely, making accurate identification more difficult [6,34,35]. In response to these documentation issues, we standardized the screening process within the EHR by using ICD-10 dementia diagnostic codes and the patient problem list to improve case identification accuracy [29,36,37]. Additionally, an automated dementia flag was added to the EHR to facilitate rapid identification of eligible patients and their CPs and to support more timely and accurate recruitment in the fast-paced inpatient environment [38]. When potential participants’ eligibility for the study could not be determined due to an unclear diagnosis of dementia, but with a diagnosis of “memory loss” or “cognitive decline,” the study team used the instant messaging feature within the EHR to swiftly contact the patient’s clinical team to clarify the diagnosis and request updated documentation. These efforts were intended to support more accurate and efficient identification of eligible dyads, which serves as a key component of recruitment in inpatient settings.

Beyond documentation and clinician collaboration, our findings highlighted the structural challenge of unpredictable CP presence during hospitalization. In inpatient settings where admissions are often unplanned and hospital stays can be brief, particularly among people living with dementia, recruitment opportunities may be constrained by narrow and fluctuating windows of CP availability. Prior work has documented the challenges of recruiting hospitalized patients living with dementia and their CPs, highlighting institutional and logistical barriers inherent to this context [39]. The timing and duration of CP visits were frequently misaligned with screening and enrollment processes, particularly given the paper-based delivery of the D-CHAT. If recruitment workflows are not responsive to these temporal constraints, eligible CPs may be missed despite accurate case identification. Strategies such as frequent bedside checks, shift-to-shift reminders, and flexible scheduling were therefore not merely logistical adjustments but efforts to align recruitment actions with rapidly changing availability patterns. These findings suggest that improving accessibility in inpatient settings may require temporal adaptability, designing workflows that can rapidly respond in real time to unpredictable CP presence.

Recruitment feasibility in inpatient settings is influenced by existing workload structures and competing clinical priorities, as clinical research staff frequently report that tensions between clinical duties and research tasks disrupt recruitment processes [40]. In high-turnover inpatient settings, additional screening and coordination tasks may be difficult to integrate sustainably into routine practice. If these workload realities are not addressed, recruitment processes are unlikely to become reliably embedded in daily operations. In this study, the research team assumed primary responsibility for screening and coordination tasks, thereby minimizing added burden on nurse leaders and frontline clinicians. This redistribution of effort may have reduced perceived participation “cost” for clinical staff and supported more consistent recruitment implementation, but it required substantial investment of study team time within narrow hospitalization windows. These trade-offs suggest that managing the “price” of recruitment involves deliberate allocation of organizational capacity rather than reliance on clinician motivation alone. Improvements in recruitment efficiency may therefore depend more on strengthening dedicated research infrastructure than on expanding clinical staff workload [41]. For long-term sustainability, inpatient recruitment efforts may benefit from formally designating specific roles or personnel responsible for screening and coordination activities, ensuring alignment between recruitment responsibilities and available resources.

The challenges of limited staff capacity and fragmented clinical–research communication highlight the importance of clinician partnership in CP recruitment. In our study setting, effective recruitment was facilitated by close collaboration with nurse leaders and other frontline clinicians (e.g., certified nursing assistants) who interact with CPs daily and are uniquely positioned to identify eligible CPs, introduce the study at appropriate moments, and foster positive perceptions and understanding of research participation among CPs [42,43,44]. Recruitment success may depend in part on strengthening clinicians’ awareness, motivation, and sense of ownership of their recruitment role. Clinicians’ buy-in—associated with perceptions of the relevance, feasibility, and value of study activities—can support the translation of recruitment procedures into consistent practice [45,46]. To support nurse leaders in this liaison role, we established standardized communication pathways to enable timely, coordinated recruitment efforts. These included weekly email updates on enrollment progress, secure email communication for real-time coordination using deidentified information, and an EHR-embedded secure chat function as an in-hospital backup. Together, these processes supported more structured information exchange, enhanced transparency regarding recruitment progress, protected patient and CP confidentiality, and strengthened collaboration between the study team and clinicians by aligning with clinical workflow demands and facilitating timely follow-up with eligible CPs.

In many inpatient settings, recruitment can be further constrained by institutional policies that limit direct researcher contact with CPs, requiring that recruitment be mediated through a member of the clinical team [6,33]. Within this constraint, study promotion needs to be intentionally designed rather than passively assumed. Our multi-pronged approach, including distributing study flyers, using clinician-guided introduction scripts, and implementing a two-step confirmation process, was structured to comply with hospital and institutional review board policies while maintaining meaningful engagement opportunities. This design leveraged the trust CPs instill in nurse leaders [47] and enabled engagement to proceed whether CPs were present in person or more easily reached by phone. The study team’s availability outside typical work hours further supported participation among CPs with demanding work and family responsibilities, including many in the “sandwich generation.” Consistent with prior research, these findings highlight the value of adaptable, policy-aligned recruitment strategies that are sensitive to institutional constraints while actively facilitating CP engagement [31].

Beyond the practical demands of recruitment, these findings underscore the broader need to embed “caregiver-centered care” into hospital workflows [48]. The concept originates from the Canadian caregiver-centered care framework, which emphasizes recognizing and supporting CPs as integral partners in care. Hospitals are often the first point of contact where CPs express concerns, encounter care complexity, and need support navigating responsibilities after discharge. Clinician engagement in recruitment—through recognizing CPs, initiating conversations, and validating their role—reflects core principles of this framework, including acknowledging CPs as part of the care team and meeting their informational and emotional needs [49]. Improving documentation, communication, and clinical-research partnerships can strengthen recruitment and equip hospitals to identify and support CPs during dementia care transitions, which helps to advance the priorities in the National Strategy to Support Family Caregivers [14].

## 5. Implications of the Study for Current Practice and Research

This study offers three context-informed considerations that may inform recruitment planning in similar inpatient settings. First, our findings suggest that effective CP recruitment may require clinical and research teams to function as a more integrated unit with shared goals, continuous communication, and a unified approach to identifying barriers and developing strategies to address challenges inherent to inpatient recruitment. Second, the usability and adaptability of the EHR appeared to play an important role in supporting recruitment in this setting. Improving EHR documentation, such as creating dedicated CP fields and integrating CP information with patient emergency contact records, may support both clinical workflows and research recruitment. These enhancements may support earlier CP assessment and may help study teams identify eligible CPs more efficiently. Third, in this study, effective CP recruitment occurred alongside the implementation of a proactive, adaptive, and multi-pronged set of recruitment strategies introduced concurrently during the study period. These strategies were developed in response to the unpredictable timing and short duration of CP presence at the bedside. Because CP availability changes rapidly and visit windows are brief, the study team, particularly in the context of a randomized controlled trial, may need to continuously adjust recruitment actions in real time. This adaptive process was only possible through close partnership with clinical staff, who helped identify emerging barriers and collaboratively develop workable solutions that fit within clinical workflows. Such real-time coordination was viewed as important for reducing missed recruitment opportunities and supporting the feasibility of trial implementation by ensuring that recruitment strategies remained responsive to the realities of hospital environments. Future directions include evaluating the sustainability of individual recruitment strategies and their long-term impact on overall recruitment performance, including whether early improvements can be maintained and integrated into routine hospital workflows.

## 6. Limitations of This Study

This study has several limitations. First, although the study employed a rigorous qualitative analytic approach, it was not designed as a formal comparative evaluation of recruitment strategies. Instead, the findings were generated from analyses of implementation meeting data, which limits the extent to which the effectiveness of individual strategies can be compared. Second, because data collection occurred only during the early implementation phase of the parent study, our ability to assess how recruitment strategies evolved and were adapted during later implementation stages was limited. Third, the analysis relied primarily on perspectives from the study team and hospital stakeholders and did not directly include CPs’ perspectives, which may limit insight into participant-side barriers, facilitators, and perceptions of recruitment efforts. Fourth, recruitment outcomes were summarized using monthly enrollment and cumulative enrollment rates, which provided a high-level view of recruitment performance but limited more granular quantification, such as CP or hospital stakeholder satisfaction or resource intensity associated with individual recruitment strategies. Fifth, conducting data collection and analysis within a single U.S. healthcare system may limit the generalizability of findings to inpatient settings with different organizational structures or implementation resources. The applicability of specific recruitment strategies may vary depending on institutional capacity, EHR infrastructure, and local regulatory requirements, and may therefore require adaptation to local contexts. Finally, the findings may be subject to study team bias or subjective interpretation despite efforts to systematically document decision-making processes.

## 7. Conclusions

Recruiting CPs in inpatient settings is challenging but achievable. The hospital environment presents unique, context-specific barriers that can hinder CP identification and delay recruitment processes. Our findings suggest that achieving strong enrollment and maintaining efficiency may benefit from a multi-pronged strategy and close collaboration with nurse leaders. Key considerations that may inform recruitment planning in comparable inpatient contexts include integrating recruitment early into admission and unit workflows, developing EHR-based screening and documentation procedures, using structured and proactive outreach scripts delivered by clinician champions, systematically tracking recruitment attempts, and offering flexible consent pathways aligned with CP availability.

## Figures and Tables

**Table 1:** Characteristics of meetings.

ID	Type of Meeting	Date of Meeting	Format	Number of Participants	Participant Roles	Duration
1	Full team	10 April 2024	Virtual	2	1 study team member; 1 unit nurse leader	18 min 19 s
2	Full team	10 April 2024	Virtual	4	2 study team members; 2 nurse leaders	23 min 39 s
3	Full team	28 May 2024	Virtual	6	2 study team members; 4 nurse leaders	32 min 16 s
4	Full team	24 July 2024	Virtual	8	3 study team members; 3 nurse leaders; 2 hospital study coordinators	35 min 49 s
5	Full team	11 September 2024	Virtual	9	3 study team members; 4 nurse leaders; 2 hospital study coordinators	46 min 27 s
6	Full team	9 October 2024	Virtual	7	3 study team members; 3 nurse leaders; 1 hospital study coordinator	44 min 01 s
7	Study team only	11 October 2024	In-Person	5	5 study team members	56 min 35 s
8	Study team only	25 October 2024	In-Person	6	6 study team members	44 min 39 s
9	Study team only	8 November 2024	In-Person	4	4 study team members	46 min 18 s
10	Full team	13 November 2024	Virtual	9	4 study team members; 4 nurse leaders; 1 hospital study coordinator	28 min 30 s
11	Study team only	6 December 2024	In-Person	6	6 study team members	37 min 09 s
12	Full team	11 December 2024	Virtual	4	2 study team members; 2 nurse leaders	23 min 45 s

**Table 2:** Recruitment barriers and corresponding strategies identified in the study, organized by Social Marketing Framework (SMF) domains with illustrative examples.

SMF Domain	Barriers	Strategies	Representative Implementation Activities
Defining and identifying the target audience	Fragmented and non-standardized EHR documentation of dementia and CP information; limited research team access to clinical information systems	Standardized EHR-based eligibility screening protocol; enhanced accuracy of dementia documentation; Research Team EHR navigation capacity-building	Apply ICD-10 coding and use the EHR problem list as the standard reference for dementia screening Confirm and clarify unclear dementia diagnoses or patient documentation with clinicians Update and maintain accurate dementia diagnoses in the EHR Request technical support and training from the hospital information technology department to improve EHR navigation Request access to specific EHR functions necessary for patient and CP screening Learn from nurse leaders’ use of EHR search functions to locate patient and CP information
Improving accessibility	Unpredictable CP availability during patients’ hospitalization	Proactive monitoring of CP presence	Conduct frequent in-person checks for CP presence on the hospital unit during regular shifts Use the EHR messaging function to remind on-duty staff to check for CP presence after regular work hours
Managing the price	Competing clinical demands and limited recruitment capacity of nurse leaders	Designated recruitment role accountability	Assign a research team member each day to conduct CP screening and enrollment Designate a primary nurse leader in each unit to lead CP recruitment and communication
Promoting the study	Institutional policy constraints on direct research engagement; low-intensity recruitment outreach mechanisms	Enhanced CP engagement and study visibility	Display study flyers in family waiting areas within the units Engage nurse leaders to initiate direct conversations with CPs to introduce the study Encourage care team members to remind CPs to review the study flyer when present on the unit
Working with partners	Inconsistent communication channels between research and clinical teams; limited frontline staff awareness and engagement	Strengthened clinician engagement and buy-in; structured research-clinical communication pathways; enhanced cross-unit nurse leader coordination	Display study flyers in staff break rooms to increase awareness of the D-CHAT among unit staff Offer educational sessions for clinicians focused on caregiving Promote the study to unit staff during professional development sessions Provide small tokens of appreciation for unit staff and nurse leaders Seek additional funding for unit-level discretionary needs Maintain regular meetings with nursing staff to address study concerns and facilitate feedback Cultivate a positive communication climate to encourage engagement Establish standardized communication channels, including weekly updates, a secure email account, and structured templates Develop a shared screening log to improve transparency and coordination in recruitment tracking Maintain a shared tracking sheet for patient admissions and CP status Coordinate recruitment efforts among nurse leaders within each unit and ensure coverage during absences Facilitate cross-unit sharing of recruitment strategies among nurse leaders

SMF, Social marketing framework; CP, care partner; D-CHAT, the Dementia Care Partner Hospital Assessment Tool; EHR, electronic health record.

## Data Availability

The datasets presented in this article are not readily available because the data are part of an ongoing study. Requests to access the datasets should be directed to the corresponding author.

## Abbreviations

The following abbreviations are used in this manuscript:

CP	Care partner
ADRD	Alzheimer’s disease and related dementias
D-CHAT	Dementia Care Partner Hospital Assessment Tool
EHR	Electronic health record
SMF	Social marketing framework
MCI	Mild cognitive decline
